# Four New Horsemen of an Apocalypse? Solar Flares, Super-volcanoes, Pandemics, and Artificial Intelligence

**DOI:** 10.1007/s41885-022-00105-x

**Published:** 2022-01-15

**Authors:** Ilan Noy, Tomáš Uher

**Affiliations:** Victoria University of Wellington, Wellington, New Zealand

**Keywords:** Solar, Volcano, Pandemic, Artificial intelligence, Extinction, D81, O33, D81, O33

## Abstract

If economists have largely failed to predict or prevent the Global Financial Crisis in 2008, and the more disastrous economic collapse associated with the pandemic of 2020, what else is the profession missing? This is the question that motivates this survey. Specifically, we want to highlight four catastrophic risks – i.e., risks that can potentially result in global catastrophes of a much larger magnitude than either of the 2008 or 2020 events. The four risks we examine here are: Space weather and solar flares, super-volcanic eruptions, high-mortality pandemics, and misaligned artificial intelligence. All four have a non-trivial probability of occurring and all four can lead to a catastrophe, possibly not very different from human extinction. Inevitably, and fortunately, these catastrophic events have not yet occurred, so the literature investigating them is by necessity more speculative and less grounded in empirical observations. Nevertheless, that does not make these risks any less real. This survey is motivated by the belief that economists can and should be thinking about these risks more systematically, so that we can devise the appropriate ways to prevent them or ameliorate their potential impacts.

## The Existential Risks we Ignore

“So in summary, Your Majesty, the failure to foresee the timing, extent and severity of the crisis [of 2008] and to head it off, while it had many causes, was principally a failure of the collective imagination of many bright people, both in this country and internationally, to understand the risks to the system as a whole.” This excerpt is from a letter written by members of the British Academy to Queen Elizabeth, in response to the question she famously posed at the London School of Economics in the aftermath of the 2008 Global Financial Crisis: Why did nobody see it coming? (Besley and Hennessy 2009).

Disasters have been the subject of social science research for some time (O’Keefe et al. 1976). Catastrophic events, ones that have the potential to extinguish the human species are also not inevitable ‘natural’ events. Indeed, the insurance term ‘Acts of God’ to describe acts that are not within the immediate power for us to prevent are mis-labelled. Disasters occur because of our actions or inactions. Epidemics are no different to earthquakes or volcanic eruptions, and their impact is also largely dependent on how we prepare and react to them. And yet, practically every country was unprepared for the COVID-19 pandemic that started in early 2020.

If economists have failed to predict or prevent the Global Financial Crisis in 2008, and the more disastrous pandemic of 2020, what else is the profession missing? This is the question that motivates this survey. Specifically, we want to highlight four catastrophic risks – i.e., risks that can potentially result in global catastrophes of a much larger magnitude than either the 2008 or 2020 events. Inevitably, and fortunately, these catastrophic scenarios have not yet occurred, so the literature investigating them is by necessity more speculative and less grounded in observations. Nevertheless, that does not make these risks any less real, any less dangerous, or any less worthy of our attention.

The four risks we examine here are space weather and solar flares, super-volcanic eruptions, high-mortality pandemics, and misaligned (or maligned) artificial intelligence. All four have a non-trivial probability of occurring and all four can potentially, and disastrously, lead to a catastrophe not very different from human extinction. As Weitzman’s Dismal Theorem suggests, the basic approach to economic assessments of risks fails for these extremely catastrophic ones (Weitzman 2009). “There is a race being run in the extreme tail between how rapidly probabilities are declining and how rapidly damages are increasing. Who wins this race, and by how much, depends on how fat (with probability mass) the extreme tails are.” (Weitzman 2011). As Weitzman points out, it is often difficult to assess how fat these tails are, because they represent sets of events that are far outside the realm of our concrete experiences. Still, this survey is motivated by the belief that economists can and should be thinking about these risks more systematically, so that we can devise appropriate ways to prevent them or ameliorate their potential impacts.

## Space Weather and Solar Flares

Active regions on the surface of the sun can produce solar flares, which are jets of solar energy and coronal mass ejections (sudden release of plasma accompanied by a magnetic field) (Kahler 1992). These phenomena are produced when local regions of the sun's magnetic field suddenly change configuration (Knipp and Biesecker 2015) and are typically interconnected, occurring within a relatively short period of time. The resulting electromagnetic radiation can create disruptions of the Earth's magnetic field (termed geomagnetic storms), commonly known as “space weather events” (Schwenn 2006).

The most important implication of these events is in terms of disruptions to electricity-powered technology. Due to the global economy’s increasing reliance on such technologies, it has grown increasingly more vulnerable to the impacts of space weather.^1^ An extended power outage that lasts for weeks or months, affecting a large population (potentially tens of millions), can easily become a major catastrophe, and even a systemic threat to society.

### Societal Impacts of Severe Space Weather and Solar Flares

Space weather events have been associated with a multitude of negative, mainly technological, direct economic consequences such as blocked radio communications, satellite damage and malfunctions, disruption to rail networks and wireless networks, and global navigation systems such as GPS (Cannon et al. 2013; Eroshenko et al. 2010). However, the most consequential possible effect is extensive damage to electricity transformers and therefore potentially long-term disruption to the electricity transmission infrastructure (Khurshid et al. 2020; Kappenman 2012). Such a disruption would cause severe problems with the supply of many basic services, including the access to pumped potable water, and the loss of all perishable foods and medications that depend on refrigeration (National Research Council 2009; Kappenman 2012).

The observed history of space weather in modern economies is very limited. The biggest geomagnetic storm of the last few decades happened in 1989 and caused transformer damage in multiple countries and an approximately 9 h-long power outages for over 6 million inhabitants of Quebec (Lakhina et al. 2004; Barnes and Van Dyke 1990).

The most severe directly observed events were the storms of 1859 (the Carrington Event) and 1921, which were roughly three times as powerful as the 1989 event and are typically considered to be a 1 in a 100-year events (Kappenman 2010). However, since economies at the time were not so dependent on electricity (especially, of course, in 1859), the impact of both these events was much less severe than the impacts of the 1989 event and consisted mostly of disruption to the telegraph service (Boteler 2006).

While these historical events are associated only with relatively mild societal impacts, a similar event today would have severe global consequences. Many of the studies attempting to analyse the future potential impacts of such an event focus on the economic effects associated with major power outages. A study by Kappenman (2010) estimates that a Carrington storm hitting the United States could put more than 200 large power transformers at risk of permanent damage and cause severe damages to the power grid, leading to long-lasting (months or potentially years) blackouts for approximately 130 million people in the US and a full recovery time of 4–10 years. The total cost of a long-term power outage affecting a significant area is estimated at USD 1–2 trillion during the first year (National Research Council 2009).

In a report for Lloyd’s, Maynard et al. (2013) points out that the duration of the power outage depends largely on the availability of spare transformers. These authors estimate that, in the worst-case scenario of no spare transformers, a Carrington-like event would lead to a power outage in the US affecting 20–40 million people and lasting 5 months. They estimate an associated economic loss of USD 0.5–2.6 trillion.^2^

Moran et al. (2014) analyse the economic impacts of space weather from a global perspective and conclude that “a severe space-weather event could be the worst natural disaster in modern history” (p. 8). Assuming a power outage lasting 1 year for an event of a magnitude between the 1989 storm and the Carrington event (with annual probability of occurrence likely to be higher than 1%), the authors conclude a major disruption of global supply chains affecting all industries and estimate a global economic loss of up to USD 3.4 trillion (5.6% of global GDP) in the first year.

Oughton et al. (2016) find that an extended power outage in the US caused by a similar space weather event would lead to global economic losses with respect to global supply chain disruptions valued at USD 0.5–2.7 trillion. Using a globally integrated economic model to account for the post-event dynamic responses of global trade, they estimate a decrease of global GDP by up to USD 1.1 trillion over a five-year period. The economic loss to the US manufacturing industry is estimated to be USD 350 billion with approximately half of the losses being indirect (roughly equally split between the losses associated with upstream and downstream disruptions to the supply chain). The losses to the US insurance industry are estimated to be up to USD 334 billion, with 90% of the losses caused by service interruption within property insurance policies.

In another paper, Oughton et al. (2017) suggest that a severe geomagnetic storm, causing a power outage for 66% of the population in the US, would create a daily economic loss of USD 41.5 billion. Approximately half of the total economic loss is estimated to be inflicted indirectly outside of the blackout zone due to supply chain disruptions; Moran et al. (2014) reach similar conclusion for the global economy.

Oughton et al. (2019) estimate an economic impact of a significant geomagnetically-induced power grid failure in the UK and distinguish between scenarios in terms of the ability to forecast the event. They find that a 1 in a 100-year event with the current level of forecasting would cause a GDP loss of GBP 2.9 billion in the UK, but an enhanced forecasting ability based on further investment in this technology would bring the loss down significantly, to GBP 0.9 billion.

In all these modelling exercises, power outages for a significant portion of the population are assumed to lead to cascading effects over many sectors. National Research Council (2009) emphasised banking and finance, government services and emergency response. Eventually, however, all economic sectors would be adversely affected (Moran et al. 2014; Riley et al. 2018). Apart from the sectoral vulnerability to power outages due to the reliance on electricity, Moran et al. (2014) note that the global economic production system today is made even more vulnerable due to the common use of practices such as just-in-time production, reduced inventories, and increased reliance on long-distance supply chains with many links.

More difficult to model, but maybe not less important to evaluate, is the public response to such an event. Hapgood et al. (2021) suggest that a power grid disruption caused by a severe space weather event would lead to panic buying and stockpiling of essential goods such as petrol, bottled water, non-perishable foods, and toilet paper. There could be various flow-on effects from this panic. Ultimately, the longer-term economic consequences of that are unclear.

It is uncontroversial that an extended power outage for a significant portion of the population would lead to disastrous economic impacts. However, the potential of space weather events of the magnitudes discussed above to cause such extended power outages are questioned by studies such as NERC (2010, 2012) and Cannon et al. (2013). They argue that extensive power grid hardware damage is unlikely, and the more probable consequence is a temporary system collapse due to voltage instability. In their view, this will result in only short-term stoppages in electricity supply and thus modest economic consequences.

### Superflares

Research focusing on the risk of even more severe space weather events is scarce, partially because there have so far been no direct observations of “superflares” in our solar system. Some evidence suggests that a superflare 100 times stronger than the Carrington event may have happened in AD 775 (Melott and Thomas 2012; Usoskin et al. 2013; Mekhaldi et al. 2015) and may have led to regional changes in the Earth’s surface temperature (Sukhodolov et al. 2017). The nature of this AD 775 event, however, is inconclusive (Cliver et al. 2014; Stephenson 2015; Neuhäuser and Neuhäuser 2015). Another extremely powerful event possibly happened in AD 993 (Miyake et al. 2013; Mekhaldi et al. 2015). Furthermore, astronomical observations of other sun-like stars in our galaxy suggest that such events are indeed possible (Maehara et al. 2012; Nogami et al. 2014).

Lingam and Loeb (2017a) propose that the most powerful superflares might have been the cause of some of the previous mass extinction events and that a very rare superflare with energy 100,000 times larger than the Carrington event might be able to destroy the ozone layer and lead to widespread destruction of ecosystems with potentially existential consequences. In a follow-up paper, Lingam and Loeb (2017b) suggest that the societal vulnerability to superflares in terms of economic damage is increasing rapidly, due to the growth of technological infrastructure. They propose a mitigation strategy of setting up a protective shield between the Earth and the Sun to avoid these dire consequences.

### Probability

In the academic literature, a Carrington-like space weather event is typically referred to as about a 1 in a 100-year event (Riley 2012; Riley and Love 2017). However, Moriña et al. (2019) find this probability of occurrence to be an order of magnitude lower at 0.46%-1.88% over a ten-year period.

For more severe space weather events, Usoskin and Kovaltsov (2012) estimate an annual probability of 0.1–1%, but for an event 100 times more powerful. Maehara et al. (2012) note that flares of such magnitude occur on sun-like stars every 800 years, similarly to Shibayama et al. (2013). While Shibata et al. (2013) further suggest that this frequency applies for the Sun as well, Karoff et al. (2016) argue that the frequency for the Sun might be lower by an order of magnitude due to the relatively lower solar activity compared to other stars. Fortunately, Lingam and Loeb (2017a) propose that an extinction-level superflare with energy levels 100,000 times larger than the Carrington event are much rarer. These might occur with a frequency of once in 20 million years.

### Conclusion

Society's vulnerability to the adverse impacts of severe space weather (such as a Carrington event) has increased dramatically during the twentieth and twenty-first centuries due to our rapidly increasing reliance on electricity, economic globalisation, and other modern economic practices (e.g., just-in-time production with long supply chains). These events now have the potential to create globally destabilizing effects and economic damages worth trillions of dollars due to the extended power outages and other effects they may initiate (such as massive migrations, or local and international conflicts over scarce resources).

Within the regions directly affected by the power outage, all economic sectors could be affected. However, adverse economic effects would also manifest outside of the directly affected areas due to global supply chain disruptions, changing demand patterns, and shifts in the location of people. These can potentially account for up to half of the total economic loss. The economic losses caused by these events may be influenced by factors such as availability of spare transformers or the level of our forecasting ability with respect to their occurrence. However, the potential of these events to create extended power outages across vast regions remain debated and require a lot more research, not only by physicists, but especially by economists and other social scientists.

## Super-volcanoes

Super-volcanic eruptions are typically defined as eruptions of magnitude 8.^3^ These volcanic explosions are tens or hundreds of times larger than eruptions observed in the recent millennia and have potential to create global or even existential catastrophic outcomes (Rampino 2008). These large eruptions are likely to generate many local and global negative consequences. After a large eruption, the surrounding area of possibly hundreds of km^2^ can be covered with falling rock and magma, a thick layer of ash can cover continental amount of land and massive amounts of volcanic ash and gases might be released into the atmosphere (Self 2006). The literature on the climatic and societal impacts of such eruptions is not as sparse as for solar flares, given the richer historical record which includes large volcanic eruptions of magnitude ~ 7. A pertinent example is the eruption of Tambora in 1815, which led to a global ‘year without a summer’ and had an estimated magnitude of 6.9); but even in this case, most of this literature is not interested in the super-eruptions that are the focus here (Choumert-Nkolo et al. 2021).

The main, global, and potentially existential threat of a major eruption lies in the adverse climatic effects it can induce. Large volcanic eruptions of magnitudes ~ 7 and higher can cause significant atmospheric cooling, lasting for years or potentially decades. These are often referred to as “volcanic winters” (Newhall et al. 2018; Self 2015, 2006; Jones et al. 2005; Gathorne-Hardy and Harcourt-Smith 2003; Handler 1989; Timmreck and Graf 2006). The global drop in temperatures is caused by the injection of volcanic ash and sulphate aerosols into the stratosphere which block the incoming sunlight. The duration of the climatic cooling may be extended due to feedback loops of ocean cooling and ocean and land ice formations further cooling the atmosphere by reflecting sunlight (Rampino and Self 1992, 1993).^4^ Super-volcanic eruptions also appear to have a negative effect on the ozone layer, increasing the amount of life-endangering ultraviolet radiation that reaches the Earth’s surface (Xu et al. 2019; Kutterolf et al. 2013).

Studies of large historical eruptions and their climatic impacts provide relevant insights. The eruption of Mount Tambora in Indonesia in 1815 was one of the biggest known eruptions in the last two thousand years, releasing approximately 30 km^3^ of magma (Self et al. 2004). While typically not considered a super-volcanic eruption, it is estimated to have caused severe climatic effects of global atmospheric cooling of 1 °C (Robock 2005; Rampino 2008; Rampino and Self 1982) and the following year has often been referred to as “the year without summer”. Major climatic impacts were also identified for possibly the strongest volcanic eruption of the last two millennia in Rinjani, Indonesia in 1257 (estimated magnitude 7) (Stothers 2000; Oppenheimer 2011; Newhall et al. 2018).

The super-volcanic eruption of Mount Toba in Indonesia around 74,000 years ago (magnitude 8) is considered one of the greatest known volcanic events in geological history. It is estimated to have produced severe and long-lasting global cooling of 3–5 °C (Rampino and Self, 1992, 1993). However, these findings about the longevity and the severity of the climatic impacts are disputed by Timmreck et al. (2010) and Lane et al. (2013).

### Economic Impacts of Super-volcanic Eruptions

Decreased temperature, sunlight, and precipitation have a negative impact on the photosynthetic ability of plants and therefore vast and cascading agricultural, ecological, environmental, and other implications (Robock 2000). Furthermore, associated cooling of the oceans can lead to the weakening of ocean circulation and produce further weather anomalies (Rampino 2008). The resulting disruption to agriculture, crop yields and global food production will be quite sudden, and is therefore considered to be the most consequential effect of large volcanic eruptions.

Severe agricultural impacts were shown to have followed the 1815 Tambora eruption. The evidence suggests that the climatic effects of a temperature drop and decreased precipitation in monsoon regions led to agricultural productivity decline and crop failures on multiple continents (Raible et al. 2016; Brönnimann and Krämer 2016; Oppenheimer 2003). This resulted in an increase in food prices and in combination with political turmoil led to famine and food scarcity along with epidemic disease spread in Europe, the US and China (Brönnimann and Krämer 2016; Oppenheimer 2003). Some observers suggest that the famine crisis led to modernizations of the agricultural sector that consequently increased long-term agricultural productivity (Brönnimann and Krämer 2016). The same observers suggest that, in China, these effects may have also led to a shift from subsistence crops to opium poppy farming because of its higher tolerance for dryer conditions and its higher cash value (Brönnimann and Krämer 2016).

Others suggest that the disruptions caused by the Tambora eruption possibly contributed to societal and economic changes characterized by social unrest, the Cholera pandemic, and the economic crisis in the US, and finally led to the emergence of the early welfare state (Wood 2014). In contrast, other researchers propose that the social and economic upheavals caused a political shift to the right instead (e.g., Post 1977). Nevertheless, these conflicting accounts suggest that the eruption was not the primary driver, but a contributing factor to social change.

For the 1257 Rinjani eruption, the evidence is obviously more tentative. Some, however, do suggest that the eruption’s climatic effects and the associated agricultural decline contributed to famine and high mortality in England and Japan and to disease spread in Europe and the Middle East (Stothers 2000; Guilett et al. 2017; Campbell 2017). Furthermore, it has been argued that this event may have contributed to the decline of the Mongol and Byzantine empires (Oppenheimer 2011; and Xoplaki et al. 2016; respectively).

Today, in contrast with the 19^th^ or thirteenth centuries, society is more exposed to a major eruption since the world is more densely populated. We are also more vulnerable because we increasingly rely more on technology, and because of the interconnectedness of the global economy (Newhall et al. 2018). A super-volcanic eruption would likely cause global food supply disruptions due to a loss of one or more growing seasons (Puma et al. 2015; Toon et al. 1997). Such a decline in food production could easily surpass the amount of stored food worldwide and lead to large-scale famine, unlike local food system shortages due to a hazard event, that could almost always be mitigated by imports from elsewhere, when such trade is permitted.^5^

Rampino (2002) notes that the Asian rice crop could be destroyed by a single night of freezing temperatures during the growing season and a 3–4 °C temperature drop would destroy the Canadian grain production. A similar destruction can also be wrought on cold-sensitive tropical vegetation (Williams 2012). In any case, the overall effect on plant life would definitely create a major ecological and environmental crisis (Self 2015). Besides famines, these dynamics can also lead to the spread of specific infectious diseases, social unrest, and violent conflict over resources (Rampino 2008; Stothers 2000).

The deposited ash layer, potentially covering a continent-sized landmass, would also negatively affect infrastructure, transportation, electricity transmission, and leave large areas with contaminated water sources (Donovan and Oppenheimer 2014; Self 2015). Atmospheric ash would severely disrupt aviation, GPS systems and other satellite services and telecommunication (Newhall et al. 2018; Self 2015). Furthermore, the disruptions to telecommunications and aviation would hinder the emergency response (Rymer et al. 2005).

### Probability

Assessments of return periods for large volcanic and super-volcanic eruptions are very difficult due to the incomplete geological records and consequently the potential to underestimate their probability (Deligne et al. 2010). With respect to magnitude 7 eruptions, there have been at least three such eruptions in the last two millennia (Plag et al. 2013). It has been estimated that on average these eruptions happen once in ~ 780 years (Deligne et al. 2010) or ~ 1,200 years (Rougier et al. 2018).

Super-volcanic eruptions of magnitude 8 or higher are fortunately much rarer. Previously, it was estimated that these events happen on average once in 100,000–200,000 years (Mason et al. 2004). However, a recent study by Rougier et al. (2018), to better account for the under-recording bias, estimates a return period of these events to be significantly lower at 17,000 years. Ord (2020) suggests that the likelihood of an existential catastrophe caused by a super-volcanic eruption within the next 100 years may be on the order of 0.01%.

### Conclusion

A large volcanic eruption of magnitude 7 would likely cause global catastrophic outcomes due to its climatic effects and consequent disruptions of global food production, infrastructure, and communication. A super-volcanic eruption of magnitude 8 or higher could potentially lead to a temporary or (in the worst case) an irreversible collapse of civilization or human extinction (Plag et al. 2013; Ord 2020). Apart from these direct effects, the volcanic-induced societal disruptions could lead to wars and conflicts and thus exacerbate or even create other existential risks.

While some scientists are exploring ways to prevent super-volcanic eruptions (Wilcox et al. 2015), currently the best approach to minimize their potential impacts seems to be monitoring, prediction and preparation of food reserves and the development of alternative food production methods (Plag et al. 2013; Denkenberger and Pearce 2016). It appears that, in the case of already identified volcanoes, super-eruptions may be predictable some time in advance (Newhall and Dzurisin 1988). However, it is likely that some super-volcanoes remain unidentified and are thus unmonitored. Plag et al. (2013) calculate that a global volcano monitoring system (with an estimated cost of USD 370 million per year) would have an expected economic benefit of at least ten times the total cost, but potentially hundreds or thousands of times. Such an estimate seems to be of even greater importance in light of the relatively recent higher frequency estimate of super-eruptions by Rougier et al. (2018).

Compared to the large historical volcanic eruptions discussed here, today’s scientists have developed a much improved ability for early detection of these events. Early warning systems are now regularly leading to pre-emptive evacuations in various volcanic hot spots (as in Indonesia or the Philippines). However, more investment is necessary, especially in areas in which large populations make emergency evacuations very challenging (e.g., for Yogyakarta or Manila).

Despite the severity of the potential outcomes, the overall risk posed by these events may still be underestimated. Large uncertainties remain. More research is needed to determine the specific climatic effects of super-volcanic eruptions and establishing which magnitudes or threshold may lead to catastrophic risk. With respect to the economic effects of large eruptions, further research of their implications for global and local economies, economic output, supply chains and the impacts on socio-economic outcomes would be valuable. Equally, research on any possible ways to ameliorate impacts (such as with food storage) should be pursued more rigorously.

## High-Mortality Pandemics

A naturally occurring pandemic (i.e., not from an engineered pathogen) that would threaten human extinction is a very small probability event. However, historical accounts point to several instances where disease spread played an important role in causing very significant decline of specific populations. For example, the introduction of novel diseases to the Native American population during the European colonization of the Americas had deadly consequences. It is difficult to distinguish the effects of the diseases that came with the Europeans from the war and conflict they also brought with them. Nevertheless, during the first hundred years of the colonization period, the American population may have been reduced by as much as 90% (Ord 2020).

Moreover, two major pandemic events, the Justinian Plague in the sixth century and the Black Death in the fourteenth century appear to have been severe enough to cause a significant population decline of tens of percent in the populations they affected. Both events are believed to have been caused by plague, an infectious disease caused by the bacteria Yersinia Pestis (Christakos et al. 2005; Allen 1979). While there is a certain degree of uncertainty involved in studying these events’ societal impacts, historical accounts in combination with modern scientific methods provide us with some valuable insights into the effects they may have had on the societies of the time.

With respect to the possibility of a future catastrophic global pandemic, it appears that this risk is increasing significantly along with the advances in the field of synthetic biology and the rising possibility of an accidental or intentional release of an engineered pathogen. While some of the scientific efforts in the field of synthetic biology are directed towards increasing our understanding and our ability to prevent future catastrophic epidemic threats, the risk stemming from these activities is non-trivial, and may outweigh their benefits.

### The Justinian Plague

The Justinian Plague severely affected the people of Europe and East Asia, though estimates of its overall mortality vary. Focusing exclusively on the first wave of the pandemic (AD 541–544), Muehlhauser (2017) suggests the pandemic was associated with a 20% mortality in the Byzantine empire. This estimate is based on the mortality rate estimated for the empire’s capital, Constantinople, by Stathakopoulos (2007) to produce a death toll of roughly 5.6 million. For a longer time span, AD 541 to 600, which included subsequent waves of the plague, scholars estimate a higher mortality rate of 33–50% (Allen 1979; Meier 2016).

The demographic changes associated with this high mortality led to a significant disruption of economic activity in the Byzantine empire (Gârdan 2020). A decline in the labour force caused a decline in agricultural production which led to food shortages and famine (Meier 2016). Trade also collapsed. Decreased tax revenues caused by the population decline initiated a major fiscal contraction and consequently a military crisis for the empire (Sarris 2002; Meier 2016). In the longer run, however, the massive reduction of the labour force appears to have had a positive economic effect for the surviving laborers, as the increased marginal value of labour caused a rise in real wages and per capita incomes. These beneficial effects for the survivors were also observed after the Black Death (Pamuk and Shatzmiller 2014; Findlay and Lundahl 2017).

The mortality and the disruption of activity the plague caused in the Byzantine empire also led to further direct and indirect cultural and religious consequences. Meier (2016) particularly highlights the plague’s indirect effect of an increase in liturgification (a process of religious permeation and internalization throughout society as defined by Meier 2020), the rise of the Marian cult, and the sacralization of the emperor.

The direct and indirect effects of the plague also appear to have had far-reaching and long-term political repercussions. The societal disruptions caused by the plague are believed to have significantly weakened the position of the Byzantine empire and arguably led to the decline of the Sasanian empire (Sabbatani et al. 2012). Interestingly, the pandemic indirectly favoured the nomadic Arab tribes who were less vulnerable to the contagion while traveling through desert and semi-desert environments during the initial expansion of Islam (Sabbatani et al. 2012).

Of note is the absence of a scientific consensus on the severity of the Justinian Plague’s impacts. For example, Mordechai and Eisenberg (2019) and Mordechai et al. (2019) argue against the maximalist interpretation of the historical evidence described above. They suggest that the estimated mortality rate of the plague is exaggerated, and that the pandemic was not a primary cause of the transformational demographic, political and economic changes in the Mediterranean region between the sixth and eighth century. Recently, White and Mordechai (2020) highlighted the high likelihood of the plague having different impacts in the urban areas of the Mediterranean outside of Constantinople.

### The Black Death

The Black Death which ravaged Europe, North Africa, and parts of Asia in the middle of the fourteenth century is considered the deadliest pandemic in human history and potentially the most severe global catastrophe to have ever struck mankind. With respect to its mortality, Ord (2020) argues that the best estimate of its global mortality rate is 5–14% of the global population, largely based on Muehlhauser (2017).

The plague created a large demographic shock in the affected regions. It reduced the European population by approximately 30–50% during the 6 years of its initial outbreak (Ord 2020). It took approximately two centuries for the population levels to recover (Livi-Bacci 2017; Jedwab et al. 2019b). As the mortality rates appear to have been the highest among the working-age population, the effects on the labour force were acute (Pamuk 2007).

The plague's mortality, morbidity and the associated societal disruption led to a major decline in economic output both in Europe (Pamuk 2007) and the Middle East (Dols 2019). In Europe, however, this decline in economic output was smaller than the decline in population; output per capita began to increase within a few years of the initial outbreak (Pamuk 2007).

The large demographic shock caused by the plague led to a shift in the relative price of labour which, similarly to the Justinian Plague, had a positive impact on wages. With a reduced labour force, real wages and per capita incomes in many European countries increased and were sustained at higher levels for several centuries (Voigtländer and Voth 2013a; Jedwab et al. 2020; Pamuk and Shatzmiller 2014). Scott and Duncan (2001) point out that real wages approximately doubled in most countries of Europe in the century following the plague.

An additional insight into the long-run relationship between the Black Death’s mortality and per capita incomes in Europe is offered by Voigtländer and Voth (2013a). Using a Malthusian model, they suggest that over time, the rise in income caused by the plague’s mortality led to an increase in urbanization and trade. Furthermore, the increased tax burden (per capita), combined with the contemporary political climate, increased the frequency of wars. Consequently, higher urbanization and trade led to an increase in disease spread which along with a more frequent war occurrence caused a long-term increase in mortality and a further positive effect on per capita incomes. In this way, the Black Death appears to have created a long-lasting environment of high-mortality and high-income specifically in Western Europe, functioning as an important contributing factor to its economic growth in the next centuries (Alfani 2020). However, while in Western Europe incomes remained elevated over the next centuries, in Southern Europe they began to decline as the Southern European population started recovering after AD 1500 (Jedwab et al. 2020).

Apart from the positive effects on wages, the increased marginal value of labour combined with other factors had further economic and social implications. A decreased relative value of land and the lack of workforce to use it effectively caused land prices and land rents to decrease (Jedwab et al. 2020; Pamuk 2007). A decreased marginal value of capital assets in general led to a lapse in the enforcement of property rights (Haddock and Kiesling 2002). Interest rates and real rates of return on assets also decreased (Pamuk 2007; Jedwab et al. 2020; Pamuk and Shatzmiller 2014; Jordà et al. 2021; Clark 2016).

Higher wages in combination with a relative abundance of land increased people’s access to land/home ownership, likely reducing social inequality (Alfani 2020). On the other end of the income distribution, decreased incomes for landowners led to an overall decrease in income inequality (Jedwab et al. 2020; Alfani and Murphy 2017).

With respect to the effects on agriculture, the structure of agricultural output moved away from cereals to other crops following the plague. Furthermore, the workforce shortages and the incentives to increase the labour supply are believed to have caused a shift from male-labour intensive arable farming towards pastoral farming, consequently raising the demand for female labour (Voigtländer and Voth 2013b). However, while the Black Death appears to have caused certain structural agricultural changes, Clark (2016) finds no effect of the plague on agricultural productivity in the long run.

In terms of other social consequences, the evidence suggests that the plague's mortality reduced labour coercion, particularly throughout Western Europe (Jedwab et al. 2020; Haddock and Kiesling 2002; Gingerich and Vogler 2021). The increased bargaining power of labour caused by the plague’s demographic shock contributed to and accelerated the decline in serfdom and development of a free labour regime. Gingerich and Voler (2021) further argue that these effects may have had long-lasting political implications and that a decline of repressive labour practices (such as serfdom) permitted the development of more inclusive political institutions. They find that the regions with the highest mortality were more likely to develop participatory political institutions and more equitable land ownership systems. They find that centuries later, In Germany, the populations in these high-mortality regions were less likely to vote for Hitler’s National Socialist (Nazi) Party in the 1930 and 1932 elections in Germany.

However, the positive effects on the emergence of freer labour did not take place in Eastern Europe, where serfdom was sustained and even intensified. Robinson and Torvik (2011) attempt to explain this asymmetry arguing that these differential outcomes may have been caused by the varying power and quality of institutions. The authors suggest that opportunities generated by the increased bargaining power of labour, in an environment of weak institutions, were less likely to lead to a positive effect than in the case of regions with stronger institutions (with more robust rule-of-law or less corrupt or predatory practices).

Apart from causing a negative demographic shock to the affected populations, the Black Death appears to have caused further indirect demographic changes, particularly in Western Europe. The increased employment opportunities for females caused by worker shortages and a higher female labour demand led to a decline in fertility rates and an increased age of marriage (Voigtländer and Voth 2013b). This demographic transition to a population characterized by lower birth rates likely helped to preserve the high levels of per capita incomes and contributed to further economic development of certain parts of Europe, enabling it to escape the “Malthusian trap” in the following centuries (Pamuk 2007). Siuda and Sunde (2021) confirm the pandemic’s effect on the accelerated demographic transition empirically, as they find that greater pandemic mortality was associated with an earlier onset of the demographic transition across the various regions of Germany.

Unfortunately, the Black Death also led to an increase in the persecution of Jews (Finley and Koyama 2018; Jedwab et al. 2019a). Interestingly, Jedwab et al. (2019a) were able to estimate that in the case of regions with the highest mortality rates, the probability of persecution decreased if the Jewish minority was believed to benefit the local economy.

It is important to highlight that the long-term repercussions of the Black Death were highly asymmetrical. While in Western Europe the pandemic appears to have led to some long-term dynamic shifts associated with increased wages, decreased inequality and a decrease in labour coercion, this was not the case for other regions. A decrease in wages was observed for example in Spain (Alfani 2020) and Egypt. In Spain, the plague's demographic impact on an already scarce population caused a long-lasting negative disruption to the local trade-oriented economy. The workforce disruption in Egypt led to a collapse of the labour-intensive irrigation system for growing crops in the Nile valley, with consequent disastrous effects on the rural economy (Alfani 2020). Borsch (2005) argues that the economic decline in Egypt caused by the Black Death “put an end to the power in the heartland of the Arab world” (p. 114) and to the impressive scientific and technological developments that came out of this region.

A consensus for an explanation of the Black Death’s varied impacts across regions, and their determinants, does not appear to exist. However, several researchers attempt to provide partial insights. For example, Alfani (2020) considers the differential outcomes to be broadly dependent upon the initial conditions in each region. More specifically, both Robinson and Torvik (2011) and Pamuk (2007) propose that the asymmetry of impacts can largely be explained by the differences in the institutional environments of the affected societies.

It is argued that the Black Death defined the threshold between the medieval and the modern ages, similarly to the way the Justinian Plague did for antiquity and the Middle Ages (Horden 2021). Furthermore, the differential long-term outcomes of the Black Death likely provided a significant contribution to the so-called “Great Divergence” between Europe and the rest of the world and the “Little Divergence” between North-western and Southern and Eastern Europe (Jedwab et al. 2020; Pamuk 2007).

From this perspective, it would seem rational to conclude that apart from causing substantial and long-term demographic, economic, political, and cultural changes, both the Justinian Plague and the Black Death likely significantly altered the course of human history.

Considering the above, it is not unreasonable to expect that a pandemic of a similar magnitude to these past catastrophes would do the same in the present day. However, what societal impacts a pandemic of similar or higher mortality would inflict in the twenty-first century has not really been the subject of any study, as far as we were able to identify. A possibility exists, given the newly developed capacity of humanity to create new pathogens, that the outcomes of a future catastrophic pandemic will be even more adverse than those of the Justinian Plague and the Black Death.

### Probability

In terms of the probability of naturally occurring pandemics, an informal survey of participants of the Global Catastrophic Risk Conference in Oxford in 2008 shows that the median estimate for a probability of a natural pandemic killing more than 1 billion people before the year 2100 was surveyed to be 5%, and the probability of such pandemic to cause human extinction was 0.05%. Ord (2020) uses a slightly broader definition of existential risk, which apart from human extinction also includes a permanent reduction of human potential. He estimates the probability of an existential risk stemming from a natural pandemic in the next 100 years to be 0.01%.

Probability estimates for a pandemic caused by an engineered pathogen are significantly higher. These can occur due to an accidental leak during gain-of-function research creating a deadly pathogen, or even because of an intentional release of an engineered pathogen by a malicious individual or group. In Sandberg and Bostrom (2008), the perceived risk of human extinction caused by an engineered pandemic is believed to be 2% and the risk of an engineered pandemic causing more than 1 billion deaths to be 10% before 2100. Ord (2020) estimates the probability of an existential risk in the form of an engineered pandemic in the next 100 years to be slightly higher at 3% (300 times higher than his estimate for a natural pandemic). Klotz and Sylvester (2014) find that the general probability of a pandemic caused by a laboratory safety failure could be as high as 27% for a period of 10 years, suggesting there is a significant risk associated with this kind of research.

### Conclusion

Severe historical pandemic events may have significantly altered the course of history and were associated with a multitude of consequential and long-lasting societal effects. The mortality and morbidity associated with these events led to demographic, economic, political, and cultural changes and these effects manifested differently over affected regions. Of note is the impact of the Black Death’s mortality on the long-run per capita incomes and its likely relevance for the relatively higher long-run economic development of Western Europe compared to other regions. If true, the further implications of this development for the historical events of the next centuries and the present-day structure of our civilization may be hard to over-estimate.

We note the absence of research on societal effects of a plausible present-day high-mortality pandemic event (both natural and engineered) that could be associated with global catastrophic or existential risks. The dynamics of these effects would almost certainly be considerably different from the ones reported for the historical pandemics of the medieval era, due to the qualitatively different state of our technology, medicine, and social organisation. The developments in the past several hundred years have arguably both increased our ability to treat pandemic risks (with modern medicine, disease surveillance, better coordination ability, and capacity to develop new vaccines and treatments) and decreased it (increased population density, more international trade and travel, and more intensive farming). However, the combined effect of these factors is most likely, on balance, favourable.

The dramatic impacts of the COVID-19 pandemic in 2020–2021, caused by a relatively low-risk-of-mortality pathogen (when compared to these past events), suggest that a high-mortality pandemic would still likely be trajectory-changing for humanity, even after accounting for the improvements mentioned above. Indeed, considering the relatively high probability estimates (especially in the case of engineered pandemics) described in the previous section, it would be of great value if future research efforts increased our level of understanding of these risks and the way we can ameliorate them.

## Misaligned Artificial Intelligence

Artificial intelligence (AI) refers to computer programs or machines which exhibit behaviour humans would perceive to be intelligent (Kaplan 2016). The significant scientific progress of the field in the last decades has caused many to wonder about the future of this technology and its societal implications. AI may create significant risks, depending on what kinds of systems we create and how we deploy them.

With respect to advances in the field of AI, considerable improvements have been made within specific narrow domains of intelligence (narrow AI) such as computer vision or natural language processing. Notable progress has also been made towards achieving a more general kind of intelligence, such that may eventually be able to employ multiple cognitive abilities, abstraction, and reasoning, as is typical for humans (Strogatz 2018; Badia et al. 2020; Silver et al. 2018). A system with this general reasoning ability is termed artificial general intelligence (AGI).

Broadly speaking, the use of AI can lead to harmful outcomes either if the AI is programmed to achieve a harmful goal, or if the AI is programmed to achieve a beneficial goal but employs a harmful method for achieving it (Future of Life Institute, n.d.; Turchin and Denkenberger 2018a). The latter case is especially relevant for AGI, as it is argued that application of such systems could lead to catastrophic outcomes without any bad intentions or development of harmful methods by its creators (Omohundro 2008).

The development of machines that may potentially become smarter and more powerful than humans could mark the end of an era characterized by humanity’s control of its future (Russell 2019). If such a powerful agent does not share our values, the result could be catastrophic (Bostrom 2014; Ord 2020). To prevent the potential disastrous outcomes of future AI, researchers argue it is crucial to align the value and motivation systems of AI systems with human values, a task that is referred to as the alignment problem (Bostrom 2014; Yudkowsky 2016; Critch and Krueger 2020). However, objectively formulating and programming human values into a computer is a complicated task. At present, we do not seem to know how to do it.

Predicting the potential catastrophic impacts of AI is made difficult by several factors. Firstly, the risk posed by AI is unprecedented and cannot be reliably assessed using historical data and extrapolation, unlike the other types of existential risks explored in this review (space weather, super-volcanoes, and pandemics). Secondly, with respect to general and super-intelligence, it may be practically or even inherently impossible for us to predict how a system more intelligent than us will act (Yampolskiy 2020). Considering the difficulty of predicting catastrophic societal impacts associated with AI, we are limited here to hypothetical scenarios of basic pathways describing how the disastrous outcomes could be manifested.

When considering potential global catastrophic or existential risks stemming from AI, it is useful to distinguish between narrow AI and AGI, as the speculated possible outcomes associated with each type can differ greatly. For narrow AI systems to cause catastrophic outcomes, the potential scenarios include events such as software viruses affecting hardware or critical infrastructure globally, AI systems serving as weapons of mass destruction (such as slaughter-bots), or AI-caused biotechnological or nuclear catastrophe (Turchin and Denkenberger 2018a, b; Tegmark 2017; Freitas 2000). Interestingly, Turchin and Denkenberger (2018a) argue that the catastrophic risks stemming from narrow AI are relatively neglected despite their potential to materialize sooner than the risks from AGI. Still, the probability of narrow AI to cause an existential catastrophe appears to be relatively lower than in the case of AGI (Ord 2020).

With respect to the global catastrophic and existential risk of misaligned AGI, much of the expected risk seems to lie in an AGI system’s extraordinary ability to pursue its goals. According to Bostrom’s instrumental convergence thesis, instrumental aims such as developing more resources and/or power for gaining control over humans would be beneficial for achieving almost any final goal the AGI system might have (Bostrom 2012). Hence, it can be argued that almost any misaligned AGI system would be motivated to gain control over humans (often described in the literature as a ‘decisive strategic advantage’) to eliminate the possibility of human interference with the system’s pursuit of its goals (Bostrom 2014; Russell 2019). Once humans have been controlled, the system would be free to pursue its main goal, whatever that might be.

In line with this instrumental convergence thesis, an AGI system whose values are not perfectly aligned with human values would be likely to pursue harmful instrumental goals, including seizing control and thus potentially creating catastrophic outcomes for humanity (Russell and Norvig 2016; Bostrom 2002, 2003a; Taylor et al. 2016; Urban 2015; Ord 2020; Muehlhauser 2014). A popular example of such a scenario is the paperclip maximizer, which firstly appeared in a mailing list of AI researchers in the early 2000’s (Harris 2018); a later version is included in Bostrom (2003a). Most versions of this scenario involve an AGI system with an arbitrary goal of manufacturing paperclips. In pursuit of this goal, it will inevitably transform Earth into a giant paperclip factory and therefore destroy all life on it. There are other scenarios that end up with potential extinction. Ord (2020) presents one in which the system increases its computational resources by hacking other systems, which enables it to gain financial and human resources to further increase its power in pursuit of its defined goal.^6^

Another possible scenario with respect to the future of AI technology is AI merging with humans and creating artificially enhanced beings. This may indeed be a likely future outcome, considering that the development of these emerging technologies is already ongoing. The potential for this to create globally catastrophic outcomes is relatively less explored. We have not been able to identify any studies focusing on it. Could this be a path that may eventually lead us to great harm?

The hypothetical scenarios discussed here demonstrate the possible pathways through which development and use of AI technology could lead to global or existential catastrophes. However, they do not inform us about concrete societal effects associated with such outcomes, or much on how one can invest in prevention. The catastrophic risk of AI appears to be so speculative and unpredictable that we were not able to identify any estimates of more specific socio-economic impacts of this technology, or any associated risk reduction costs and benefits.

Estimating the likelihood of a catastrophic AI scenario is equally fraught (Armstrong and Sotala 2015). At present, estimates are predominantly based on expert opinion surveys. In these, experts estimate that the probability that an AI system will be capable of performing most human tasks by 2040–2050 to be around 50% (Müller and Bostrom 2016). Similarly, a survey of Grace et al. (2018) suggests a 50% probability of AI systems outperforming humans in all tasks by 2063. The respondents in Müller and Bostrom (2016) predict that such systems would become super-intelligent less than 30 years after their development, with an estimated chance of one in three that machine super-intelligence will be bad or extremely bad for humanity. A survey described in Turchin (2019) suggests that there is an 10% chance of AGI appearing relatively early in the next 10–15 years. By combining this polling with extrapolations of current technological progress, Turchin (2019) estimates that AI capable of causing global catastrophic outcomes (narrow AI included) could be possible as early as 2030–2040.

When questioned directly about existential risk from AI, an informal survey of participants in the Global Catastrophic Risk Conference in Oxford in 2008 indicated that the risk of human extinction due to a super-intelligent AI before 2100 may be as high as 5%; the highest probability out of all those considered in that event (Sandberg and Bostrom 2008). Ord (2020) puts the probability of an AI-caused existential catastrophe even higher at 10% within the next 100 years. Sandberg and Bostrom (2008), Turchin and Denkenberger (2018c) and Ord (2020) all suggest that the existential risk probability of AI is the highest out of the risks described herein.

## Conclusion

The previous section argued that Artificial Intelligence (AI) systems most likely pose the highest global catastrophic and existential risk to humanity from the four risks we described here, including solar-flares and space weather, engineered and natural pandemics, and super-volcanic eruptions. While the risk assessments are difficult for all these four hazards, the difficulty of predicting the potential disastrous dynamics of AI is most acute, even as the risk is also likely the highest.

AI may also be very relevant for the other existential risks we described here. Counter-intuitively, maybe, AI may potentially assist us in decreasing the risk of other global catastrophic and existential events such as those associated with a nuclear war, an engineered pandemic, or a solar-flare (Liu et al. 2018; Akiyama 2021; Boulanin 2019; Bostrom 2002). There are undeniably tremendous benefits in well-aligned AI systems in general, but also particularly in terms of their capabilities to reduce existential and other risks (Bostrom 2003b; Yudkowsky 2008) or increase our resilience to these shocks.^7^ We should note that AI systems pose other less extreme risks, and some observers view these are more concerning than the extinction risk we focus on here (e.g., Acemoglu 2021; Lu and Zhou 2021).

The potential impacts of the other catastrophic risks explored in this paper may generally be better understood than the future of AI, partially because they have historical precedents. We still need further inquiry and deeper insights into the differential impacts they would all likely have on any modern society or economy. Afterall, our societies today are different in many ways than the societies that were affected, for example, by the 1918 influenza pandemics, and even more when considering earlier pandemic events. Though these differences may both reduce or increase the risks we have analysed here. When we learn from the past, we also need to take these changes into account. But more generally, it is exactly the uncommonness of these risks that make them difficult to study, and difficult to mobilise the resources required to adequately prepare for them (Wiener 2016).

Apart from analysing the economic consequences of catastrophic and existential risks, some economists have attempted to explore other economic dimensions of these risks related to, for example, decision-making regarding mitigation policy (Martin and Pindyck 2015), risk insurance (Pindyck and Wang 2013) or economic evaluation of potentially catastrophic technology (Posner 2004). However, given the potential importance of the economics of global catastrophic risks, it is still the case that the topic remains under-explored.

We also note that there are, possibly, other existential risks that we have not covered in this paper – for example, nuclear war, or a catastrophic asteroid impact that can change the climate in such a way to make much of the earth uninhabitable for a significant period of time. There have been some attempts to quantify this latter risk (e.g., Reinhardt et al. (2016) and Baum (2018) and there have also been explorations of ways to ameliorate it (e.g., Baum 2019). Our choice, ultimately, was to focus on the four risks we deem most significant, but of course this decision should be questioned, and re-evaluated repeatedly, as circumstances change, and new risks potentially emerge.

The aim of this short survey was not necessarily to provide answers to the many questions that seem to arise once we start to contemplate the economic dimensions of these risks. It seems undeniable that we are currently under-investing in thinking about these risks, not to say planning for them, or developing the systems that might be necessary to prevent some of the catastrophic scenarios described here from transpiring.

The letter of the British Academy to the Queen ends with a hopeful promise. “Given the forecasting failure at the heart of your enquiry, the British Academy is giving some thought to how…[it] might develop a new, shared horizon-scanning capability so that you never need to ask your question again…. The events of the past year have delivered a salutary shock. Whether it will turn out to have been a beneficial one will depend on the candour with which we dissect the lessons and apply them in future.” The COVID-19 pandemic has clearly shown that, collectively, and arguably specifically in Britain, we have not fully learned these lessons. The only aim of this survey is to highlight this gaping holes in our understanding of the economic dimensions of the risks we face, with the hope that we will soon enough start to plug them.

## Data Availability

Not applicable (no data included).

## Footnotes

1 For example, while the 1998 five-week power outage in Auckland, New Zealand was not caused by a space weather event and was limited in its scale to inhabitants of a single city, it demonstrated the severity of the consequences that a large-scale collapse of the power grid may induce. The Auckland power outage was caused by a failure of power cables (Rennie et al. 1998).

2 The data and methodology on which this estimate is based are not disclosed.

3 A volcanic explosion’s magnitude is typically measured by the Volcanic Explosivity Index (VEI). It ranges from 0 (a non-explosive eruption) to 8, based mainly on the volume of erupted material (Newhall and Self 1982).

4 This is exactly one of the most plausible global warming feedback loops, reversed.

5 In the past, food shortages affecting multiple regions were also sometimes accompanied by prohibitions on exports (and sometimes even by stockpiling by exporting countries).

6 A different example is offered by Wiblin (2017): A misaligned AGI is tasked with inventing an anti-cancer drug, which ends up killing humans before they grow old enough to develop cancer.

7 For very useful definitions of the term ‘resilience’ across different social sciences, and specifically for macro- and micro-economic assessments, see Martin and Sunley (2015) and Rose (2017).
